# Change in and Long-Term Investigation of Neuro-Otologic Disorders in Disaster-Stricken Fukushima Prefecture: Retrospective Cohort Study before and after the Great East Japan Earthquake

**DOI:** 10.1371/journal.pone.0122631

**Published:** 2015-04-07

**Authors:** Jun Hasegawa, Hiroshi Hidaka, Shinichi Kuriyama, Taku Obara, Ken Hashimoto, Yutaka Tateda, Yuri Okumura, Toshimitsu Kobayashi, Yukio Katori

**Affiliations:** 1 Department of Otorhinolaryngology, Soma General Hospital, Soma, Fukushima, Japan; 2 Department of Otorhinolaryngology-Head and Neck Surgery, Tohoku University Graduate School of Medicine, Sendai, Miyagi, Japan; 3 Department of Disaster Public Health, International Research Institute of Disaster Science (IRIDeS), Tohoku University, Sendai, Miyagi, Japan; 4 Department of Molecular Epidemiology, Environment and Genome Research Center, Tohoku University Graduate School of Medicine, Sendai, Miyagi, Japan; 5 Division of Molecular Epidemiology, Department of Preventive Medicine and Epidemiology, Tohoku Medical Megabank Organization, Tohoku University, Sendai, Miyagi, Japan; Hamamatsu University School of Medicine, JAPAN

## Abstract

On March 11, 2011, Japan’s northeast Pacific coast was hit by a gigantic earthquake and subsequent tsunami. Soma City in Fukushima Prefecture is situated approximately 44 km north of Fukushima Daiichi Nuclear Power Plant. Soma General Hospital is the only hospital in Soma City that provides full-time otolaryngological medical care. We investigated the changes in new patients from one year before to three years after the disaster. We investigated 18,167 new patients treated at our department during the four years from April 1, 2010 to March 31, 2014. Of the new patients, we categorized the diagnoses into Meniere’s disease, acute low-tone sensorineural hearing loss, vertigo, sudden deafness, tinnitus, and facial palsy as neuro-otologic symptoms. We also investigated the changes in the numbers of patients whom we examined at that time concerning other otolaryngological disorders, including epistaxis, infectious diseases of the laryngopharynx, and allergic rhinitis. The total number of new patients did not change remarkably on a year-to-year basis. Conversely, cases of vertigo, Meniere’s disease, and acute low-tone sensorineural hearing loss increased in number immediately after the disaster, reaching a plateau in the second year and slightly decreasing in the third year. Specifically, 4.8% of patients suffering from these neuro-otologic diseases had complications from depression and other mental diseases. With regard to new patients in our department, there was no apparent increase in the number of patients suffering from diseases other than neuro-otologic diseases, including epistaxis, and allergic rhinitis. Patients suffering from vertigo and/or dizziness increased during the first few years after the disaster. These results are attributed to the continuing stress and tension of the inhabitants. This investigation of those living in the disaster area highlights the need for long-term support.

## Introduction

On March 11, 2011, Japan’s northeast Pacific coast was hit by a sudden, gigantic earthquake and subsequent tsunami. Nearly 20,000 people were killed or are still presumed missing [1]. In Fukushima Prefecture, the resultant accident at Fukushima Daiichi Nuclear Power Plant forced approximately 160,000 inhabitants to abandon their land and homes, and evacuate to other areas. Three years later, these evacuees still live as refugees far from their homes. Activities including fishing, agriculture, and farming have been limited, and many companies have been forced to stop or reduce their business operations. The anxiety and stress of the evacuees is immeasurable.

Soma City in Fukushima Prefecture has a population of 38,000 people, and is situated on the Pacific coast approximately 44 km north of Fukushima Daiichi Nuclear Power Plant. In addition to sustaining massive damage after the great earthquake and tsunami, Soma City was also affected by the release of radioactive substances from the nuclear power plant. Therefore, people have been living in a state of fear and anxiety for a long time [2].

**Fig 1:**
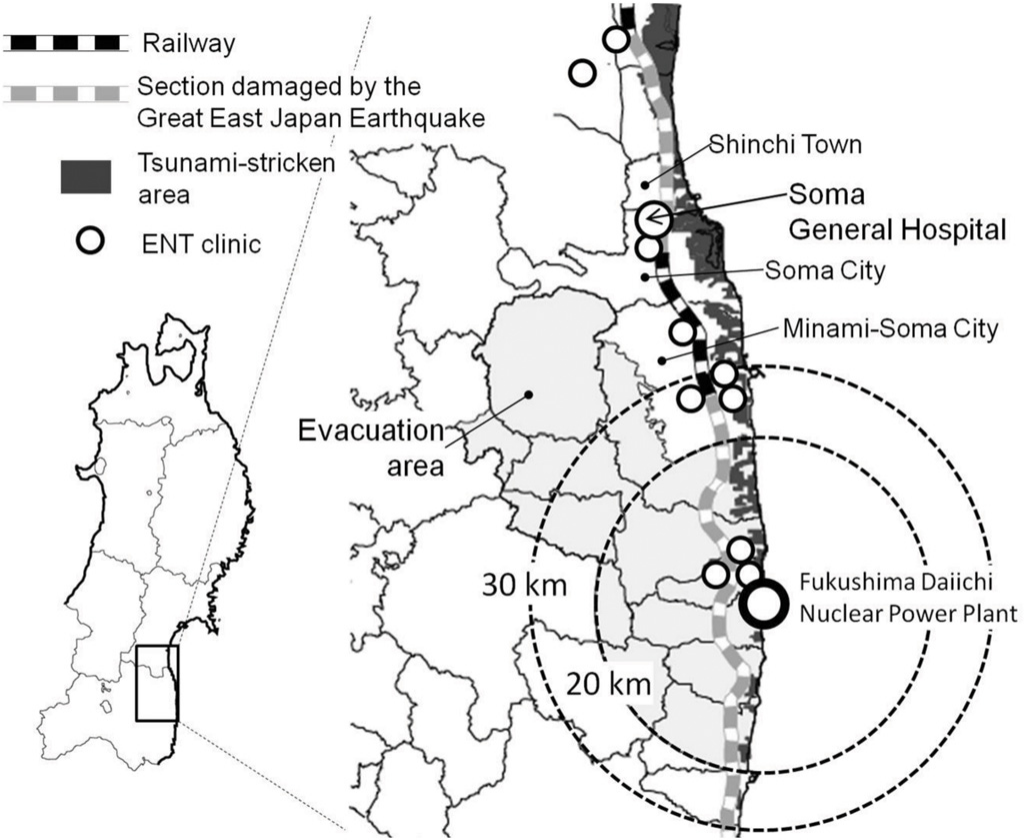
A map of the area around Soma General Hospital. Soma City is located 44 km north of Fukushima Daiichi Nuclear Power Plant. Soma General Hospital and a clinic in Minami-Soma City are the only two places that employ a full-time ENT doctor. In other hospitals, part-time doctors see patients in outpatient settings once or twice a week. Approximately one-third of the southern side of Minami-Soma City is part of the evacuation area. People in the other two-thirds of the city are suffering from a great deal of anxiety and stress. Railways were damaged by the disaster. The railway north of Soma City was washed away by the tsunami, and train service along the railway south of Minami-Soma City has been suspended for three years because of the accident at the nuclear power plant.

Our hospital is the only hospital in Soma City that provides full-time otolaryngological medical care. The other hospitals have only part-time otolaryngeal examination programs or private clinics. Thus, almost all patients who require hospitalization for ear, nose, and throat (ENT) care were referred to our department from the local district before the disaster. The accident divided the traffic network into zones north and south of the power plant. In the northern zone, our hospital is the nearest to the power plant and the only one with a full-time ENT doctor. Therefore, our facility presumably reflects well the influence of the disaster on diseases related to otorhinolaryngology (Fig 1). One possible explanation for the large outbreaks of dizziness after major earthquakes is that psychological stress causes equilibrium disturbance [3]. However, no previous reports addressed this topic from areas near the mandatory evacuation zone established to prevent high-level radiation exposure. We thus investigated the influence of the disaster on internal ear diseases that were caused by stress related to the disaster, comparing them with other otolaryngeal diseases.

## Methods

We investigated 18,167 new patients that were treated at Soma General Hospital during the four years from April 1, 2010 to March 31, 2014 (one year before and three years after the disaster). New patients were defined as those who had not visited the department in at least six months.

Of the new patients, we categorized the diagnosis of each case whose major complaints were neuro-otologic symptoms as follows:

1. Meniere's disease (MD): the cases where, together with vertigo, there was a threshold increase of bone and air conductions by 125–500 Hz in either or both of the ears in the standard pure-tone audiometry, or the cases where, regardless of dizziness, there was a repetitive change of audibility in either or both of the ears [4].
2. Acute low-tone sensorineural hearing loss (ALHL) was defined according to the criteria suggested by the Acute Severe Hearing Loss Study Group, the Ministry of Health, Labour and Welfare of Japan in 2011; namely, acute or sudden onset of cochlear symptoms including ear fullness, tinnitus, and low-tone hearing loss without vertigo. The audiometric criteria is as follows: 1) the sum of hearing levels at low frequencies of 125, 250, and 500 Hz is 70 dB or more, 2) the sum of hearing levels at high frequencies of 2, 4, and 8 kHz is 60 dB or less [5].
3. Vertigo: cases of vertigo or dizziness without hearing change.
4. Sudden deafness was diagnosed according to criteria from a previous study [6]: (a) the patients had sudden-onset sensorineural hearing loss regardless of vertigo, (b) the cause of hearing loss was unknown, (c) the hearing loss did not fluctuate, (d) the arithmetic mean of the hearing levels at 250, 500, 1000, 2000, and 4000 Hz was ≥ 40 dB.
5. Tinnitus: the cases where ear ringing was the major complaint without any subjective symptom such as vertigo or dizziness, hearing loss, and there was no apparent acute sensorineural deafness.
6. Facial palsy: peripheral facial palsy, Bell’s palsy, Ramsay Hunt syndrome, and zoster sine herpete were all included [7].

Among patients with these neuro-otological symptoms, we excluded patients with apparent disorders in the nerve center, retrocochlear tumor, middle ear disorders and cardiovascular diseases based on MRI or CT findings because these could be causes for vertigo.

We also investigated the changes in the numbers of patients whom we examined that had other otolaryngological diseases, including epistaxis, laryngopharyngitis, acute tonsillitis, peritonsillitis or abscess, and allergic rhinitis, conjunctivitis, or pharyngitis.

This study is a retrospective study of data obtained from clinical charts that were anonymized prior to analyses.

Differences in the population or diagnoses of each disease before and after the disaster were statistically examined using Dunnett’s test. All statistical analyses were conducted using SAS software (SAS, Cary, NC, USA).

All parts of the present study were performed in accordance with the guidelines of the Declaration of Helsinki. Although written informed consent was not obtained from each of the subjects, we conducted a medical interview with regard to the possibility of reporting medical records without including private information. The ethics committee of Soma General Hospital approved this study and use of oral consent.

## Result

### Social situations in the surrounding areas

**Fig 2:**
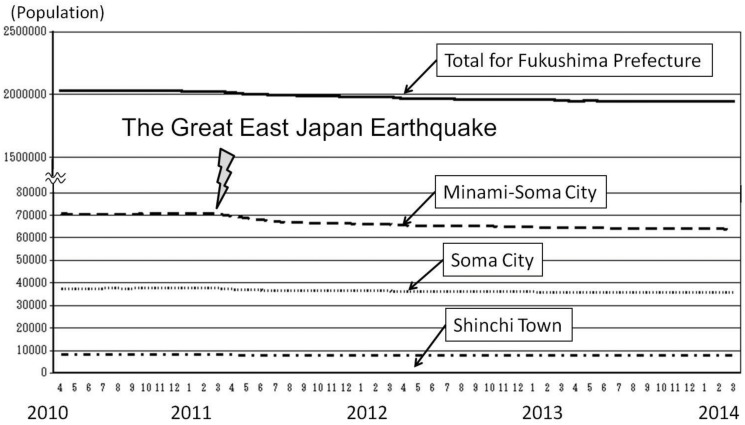
Demographic transition in Soma City, Minami-Soma City, and Shinchi Town (excerpt from Fukushima Prefectural Home Page: http://www.pref.fukushima.lg.jp/). After the disaster, many people voluntarily evacuated to other places without changing their residence registry. The exact number of those refugees has not been counted by the local governments as yet, but the population decreased by 4.4% in Fukushima Prefecture, 5% in Soma City, 9.6% in Minami-Soma City, and 6.9% in Shinchi Town.

Immediately after the disaster, victims whose socio-economic status had worsened (i.e. those who lived in the evacuation zone, whose houses were destroyed, whose breadwinners were dead or missing, or who were deprived of their work places) were exempt from paying medical expenses [8]. Since October 2012, the Fukushima Prefecture government has been managing a free health care system for inhabitants who are 18 years of age or younger, in an attempt to encourage people to remain in the prefecture [9]. However, the population of Fukushima Prefecture is still declining (Fig 2 [9]). Most of our patients live in Soma City, Minami-Soma City, or Shinchi Town. In Fig 2, changes in population of these three municipalities are also compared before and after the earthquake. The number of citizens who voluntarily evacuated to other safe places while maintaining their residence registry in this area is still unknown, but after the disaster (from April 2010 to March 2014), the overall population of Fukushima Prefecture decreased by 4.4% (2,032,302 to 1,943,414, n = 88888). Focusing on the neighboring municipalities, these decreases are 5% (37,549 to 35,648, n = 1901), 6.9% (8,278 to 7,704, n = 574), and 9.6% (70,658 to 63,871, n = 6787) in Soma City, Shinchi Town, and Minami-Soma City, respectively. Statistically, the populations of Fukushima Prefecture, Soma City, Shinchi Town, and Minami-Soma City declined significantly during the four years (Dunnett’s test, *p* < 0.05).

### Overall changes in number of patients

**Fig 3:**
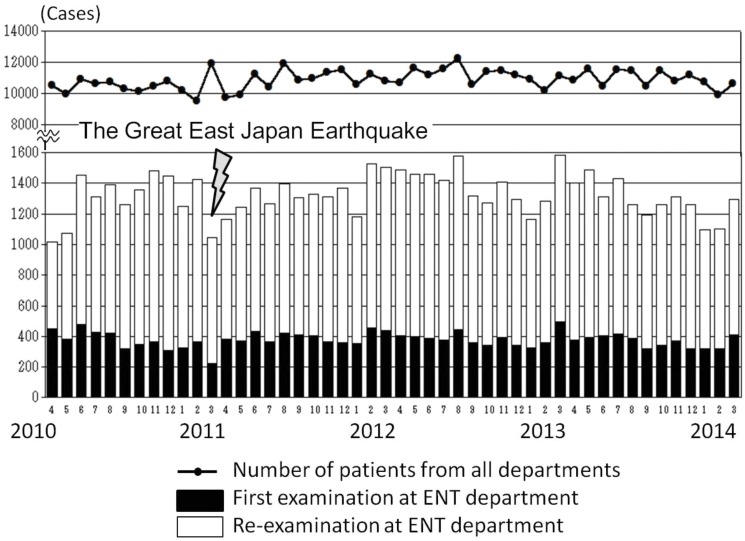
Number of outpatients from all the departments at Soma General Hospital, and the number of new patients and revisiting patients of the ENT department. Those patients who had not visited us for more than 6 months were regarded as new patients. After the disaster, the number of new patients increased 7.7% in the first year, 4.8% in the second year, but decreased 0.8% in the third year. Thus, the differences were small.

**Fig 4:**
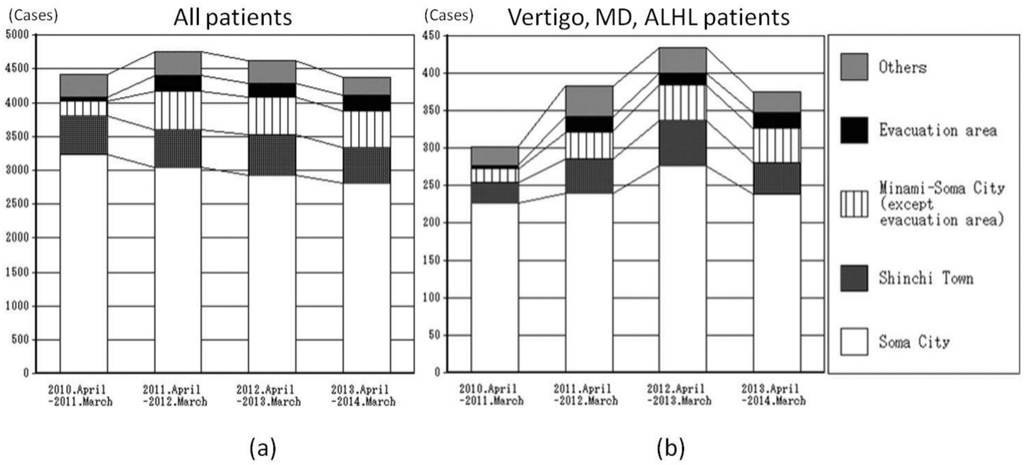
Investigation of the new ENT patients from April to the following March by living area. (a) For all new patients, the number from Soma City decreased, but that from Minami-Soma City and other evacuation areas increased. (b) Those with vertigo, MD, and ALHL increased in each area. In Soma City and Shinchi Town, the number peaked in the second year and showed a slight decrease in the third year. On the other hand, the number of new patients in Minami-Soma City and the evacuation areas remained about the same.

According to the statistics for the past 4 years, there are no remarkable changes in the total number of patients (Dunnett’s test, *p* < 0.05) (Fig 3). Focusing on the ENT department, the numbers of new patients (pre-disaster n = 4412) increased by 0.77% (n = 4753), 4.8% (n = 4625), and decreased by 0.8% (n = 4377) in the first, second, and third year after the disaster as compared with the number before the disaster, respectively. Namely, the total number of new patients in the ENT department did not change remarkably during the 4 years (Figs 3 and 4a).

### Characteristics of patients suffering from neuro-otologic symptoms

**Fig 5:**
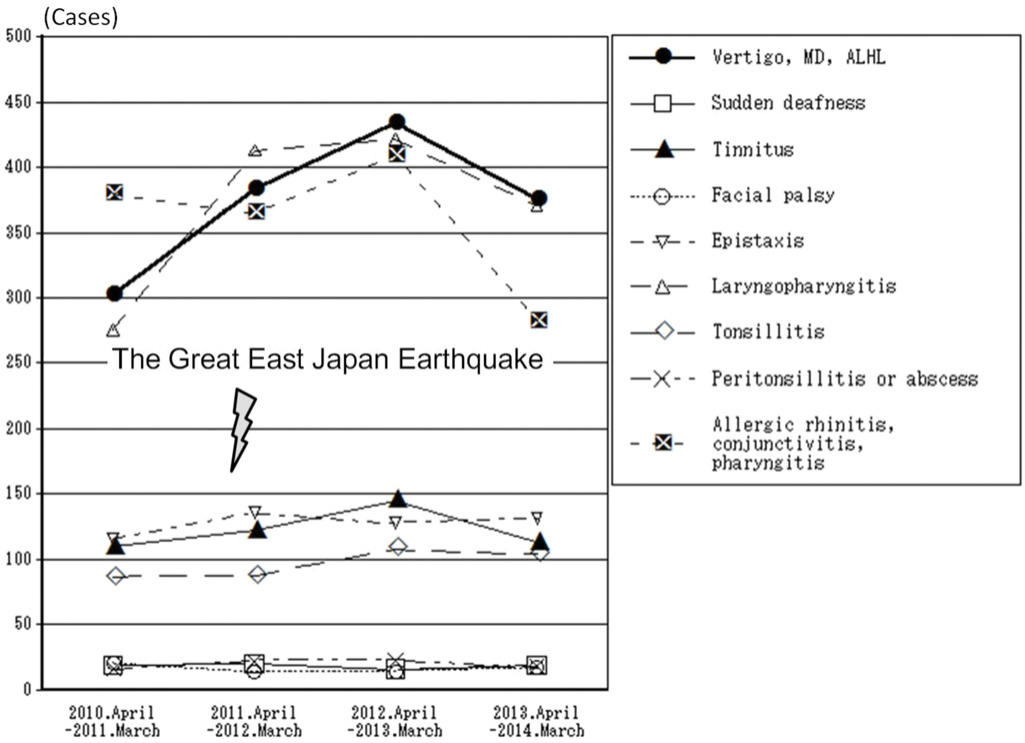
Diseases of the new patients in the ENT department of Soma General Hospital. There was an increase in the number of cases of vertigo, MD, ALHL, and laryngopharynx inflammation. However, we found no change in the number of cases of epistaxis, acute tonsillitis, peritonsillitis, parapharyngeal abscess, tinnitus, sudden deafness, facial nerve paralysis, and allergic rhinitis, conjunctivitis, or pharyngitis.

Regarding new patients, the total number of cases of vertigo, MD, and ALHL was 1,495 (1060, 341, and 94 cases, respectively). The ratios of post-disaster to pre-disaster (n = 302) cases are 1.268 (n = 383), 1.440 (n = 435), and 1.242 (n = 375) in the first, second, and third years, respectively. All of the three years after the disaster had statistically significant differences (Dunnett’s test, *p* < 0.05) compared with that of the pre-disaster year (Figs 4b and 5). Specifically, there was a gradual increase just after the disaster, a leveling off in the second year, and a slight decrease in the third year (Figs 4b and 5).

Focusing on each municipality, the number of these patients in Soma City (pre-disaster n = 226) increased by 6.2% (n = 240), 22.6% (n = 277), and 5.8% (n = 239) in the first, second, and third years, respectively. These differences were not statistically significant (Dunnett’s test). In Shinchi Town (pre-disaster n = 28), the number increased by 64.3% (n = 46), 114.3% (n = 60), and 46.4% (n = 41), respectively. Among the three years, only the number for the second year showed significant differences as compared with that of the pre-disaster year (Dunnett’s test, *p* < 0.05). In the case of Minami-Soma City, except the evacuation area (pre-disaster n = 19), the number increased by 84.2% (n = 35), 152.6% (n = 48), and 142.1% (n = 46), respectively. Although the first year had no significant differences, the second and third years had significant differences (Dunnett’s test, *p* < 0.05) (Fig 4b). Regarding the evacuation area, the total number of patients (pre-disaster n = 50) increased 4.64 times (n = 232), 4.24 times (n = 212), and 4.54 times (n = 227) compared with the number before the disaster, respectively (Fig 4a). However, the numbers of patients with vertigo, MD, or ALHL (pre-disaster n = 3) became 7 times (n = 21), 5 times (n = 15), and 7 times (n = 21) as large on a yearly basis, respectively. In terms of statistical analysis, the numbers of the first and third year had significant increases (Dunnett’s test, *p* < 0.05) as compared with those of the pre-disaster year (Fig 4b, as shown by the subtitle above the figure). These values did not decrease throughout the three years after the disaster for patients from Minami-Soma City and the mandatory evacuation zone (Fig 4b).

The numbers of new patients categorized as having tinnitus, sudden deafness, and facial palsy were relatively small and did not exhibit any remarkable changes (Fig 5).

### Characteristics of patients with other non-neuro-otologic disorders

Except for those categorized as having acute laryngopharyngitis (Dunnett’s test, *p* < 0.05), there was no apparent increase in the number of new patients in our ENT department suffering from non-neuro-otologic diseases, including epistaxis, acute tonsillitis, peritonsillitis or abscess, and allergic rhinitis, conjunctivitis, or pharyngitis (Dunnett’s test, *p* > 0.05) (Fig 5).

### Mental diseases complicated with neuro-otologic diseases

**Fig 6:**
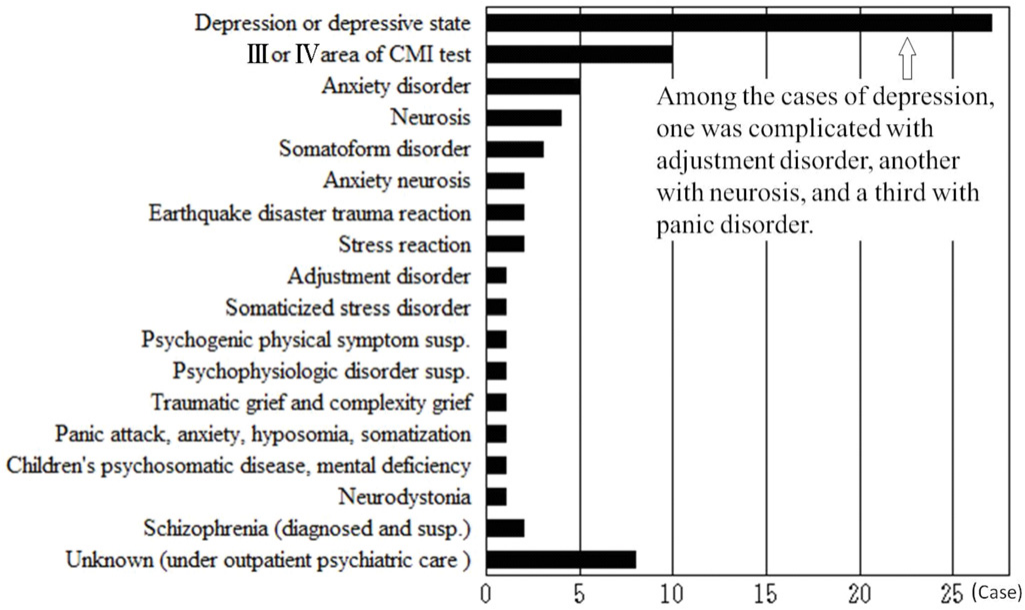
Diseases complicated with vertigo, MD, and ALHL. Depression and depressive type were the two diseases with the largest number of patients (27 cases). Other frequently seen diseases were anxiety disorder, neurosis, somatoform disorder, anxiety neurosis, adjustment disorder, and post-traumatic stress disorder (PTSD).

Before the disaster, only 12 of 302 cases (4%) suffering from neuro-otologic diseases were complicated with mental diseases. Conversely, this number increased to 28 of 383 (7.3%) in the first year, 12 of 435 (2.8%) in the second year, and 21 of 375 (5.6%) in the third year. Of these cases complicated with mental diseases, the most frequent disease was depression (27 cases), followed by somatoform disorder, neurosis, adjustment disorder, panic disorder, anxiety disorder, neurosis anxiety, and stress reaction (Fig 6).

## Discussion

To date, only a few reports have investigated outbreaks of vertigo or dizziness after disasters such as major earthquakes. There is a growing body of literature describing psychological and physical stress-related effects amongst survivors of strong earthquakes. A prospective study revealed that the quality of life of 1,756 Taiwan earthquake (magnitude 7.3; Richter scale) survivors varied according to the clinical progress of post-traumatic stress disorder (PTSD) [10]. Moreover, after the 2002 Georgia earthquake of magnitude 6 on the Richter scale, the amount of patients with vestibular symptoms increased during the initial weeks following the earthquake [11]. Our report is unique in that the number of ENT patients at a core hospital in a disaster area were compared between the pre-disaster and post-disaster periods, including a long-term investigation during the three years after the disaster.

Although the causes for MD and ALHL are still unknown, the influence of increased tension and stress has been considered to play an important role [12, 13]. The increases in patients suffering from these diseases revealed in the present study might be due to increased tension and stress of the inhabitants in the surrounding municipalities.

As for the cases categorized as having vertigo, we found nystagmus in 28% of them and diagnosed them with inner ear vertigo; 7% of them were diagnosed as benign paroxysmal positional vertigo (BPPV), judging from the nature of the nystagmus. However, we were unable to establish the neuro-otologic pathogeneses in the remaining 72% of those categorized as having vertigo because subjective symptoms, nystagmus, and hearing loss had already disappeared or were very mild, though most of them didn’t contradict the progress of inner ear vertigo in their medical history. The causes of inner ear vertigo are still unclear. Except for the abnormal otolithic organ in cases of BPPV [14], and from infection in the case of vestibular neuronitis [15], the influence of stress is suggested as a cause of inner ear vertigo [16, 17]. Vestibular symptoms of MD are also thought to be a cause [4]. Although the pathogenesis of inner ear vertigo is often unclear, there may have been cases of psychogenic dizziness [18], phobic postural vertigo [19], and equilibrium dysfunction after a major earthquake [3]. These cases seemed to have arisen from disturbance of the inner ear, as well as their individual vulnerability to anxiety enhanced by repetitive exposure to aftershocks [3].

This investigation does not include patients with repeated vertigo who revisited the ENT department. Because of the disaster and nuclear power plant accident, some patients moved out of this district, while some moved in from other evacuation areas. Therefore, the population after the disaster might not be the same as that before the earthquake. However, our department is the only ENT facility available to residents in Soma City and Shinchi Town, and almost all patients suffering from otolaryngeal diseases were likely to have come here. Secondly, compared to other diseases, there was an increase in the number of new patients categorized as having vertigo, MD, and ALHL in these two municipalities (Fig 5). Additionally, victims were exempt from paying medical expenses after the disaster [8, 9]. Under this policy, one might expect the number of patients of other diseases to also increase; however, only the number of patients with vertigo, MD, and ALHL increased.

The patients’ histories suggest that these ENT diseases can be partly attributed to the increased stress and continuation of tension and stress. During the first year after the disaster, most of the patients were directly affected by the disaster, having lost either their houses or family members. As reconstruction proceeded, cases due to the change in the living environment seemed to increase. Patients complained, saying, “I lost my relatives”, “I moved to a temporary housing unit, but I can’t fit in”, “I lost my job”, or “I have a sick family member but I can’t take care of him.” More and more patients were suffering from continued mental anxiety and stress within their new community. Some senior citizens and those weakened physically or mentally tended to suffer from stress more severely. Patients from the evacuation area near the power plant continued to suffer from MD and ALHL because they lived in temporary housing or a rented house and lived with the incessant stress of not being able to return to their hometown (Fig 4b).

In treating vertigo and MD here in Soma City, the mere administration of an anti-vertigenous drug or advice to keep calm and to have a sound mind did not help these patients improve. We had to try and not only treat their diseases but also understand their anxiety and stress.

There were some difficult cases where ENT treatment alone did not work, presumably because these cases were complicated with some mental diseases (Fig 6). Many patients with MD suffer from depression [20]. Our results suggest that there were more cases complicated with mental diseases after the disaster. In some serious cases, we used the Cornel medical index (CMI) test [21] and referred the patients to psychiatric care or prescribed psychosomatic medicine. Some patients refused our advice and hesitated to attend a psychiatric clinic or to admit that they had a mental problem. After the disaster, the number of more difficult cases greatly increased. Some were new patients but others had relapsed and chose to revisit us.

On the other hand, sudden deafness and MD are sometimes difficult to distinguish at the initial visit. Out of 122 cases of sudden deafness, hearing level in 48 cases (39%) changed over the long term, so the diagnosis was modified to MD. In this investigation, although cases of vertigo, MD, and ALHL increased in number, those of sudden deafness did not. We were unable to find any clear cause-and-effect relationship between sudden deafness and the disaster (Fig 5). The etiology and pathogenesis of MD is endolymphatic hydrops [22], but the etiology and pathogenesis of sudden deafness remain unknown. Proposed theories as to the cause of sudden deafness include vascular occlusion, tearing of the labyrinthine membranes, immune-mediated mechanisms [23], abnormal cellular stress responses within the cochlea [24], and viral infection [25] or reactivation of herpes zoster virus [23], which are suggested to be influenced by stress hormone due to a physical disorder or fatigue. Thanks to the evacuation centers and temporary housing that were quickly prepared soon after the disaster, victims were comparatively less apt to be physically fatigued. This might explain why there was no increase in cases of sudden deafness. The same thing can be said about facial palsy (Fig 5) [7].

While victims in the disaster area seem to be achieving peace and calmness in temporary housing, their state of tension and stress continues as reconstruction slowly progresses. The number of reported cases of heart disease and brain infarction have increased in the devastated area [26]. Anxiety and tension have led to peptic ulcer diseases [27]. Lack of motivation has led to a lack of exercise. Diabetes, osteoporosis, and psychiatric illnesses were feared to have worsened, especially among senior citizens [2, 28, 29].

During the three years after the disaster, the number of disaster-related deaths exceeded that of direct deaths, amounting to 1657, and the number of disaster-related suicide attempts has also increased in Fukushima Prefecture [30]. Thus, in an area such as Fukushima, where the aftermath of natural and nuclear disasters lingers, not only the victims directly affected by the disaster but also the whole population suffer from continuous anxiety and stress. Thus, we must pay attention not only to acute symptoms that appear immediately after the disaster but also to chronic symptoms that emerge in the long term. Doctors have to make careful observations, understand patients’ stress from their physical symptoms, ask about their conditions, and listen to them intently. A quick, adequate response and treatment are indispensable.

One of the limitations of this study is in calculating the number of new patients without consideration of the population that moved to different places. More than 80,000 people in the evacuation zone and 136,000 people in all of Fukushima Prefecture still continue to live uneasily as refugees, even three years after the Great East Japan Earthquake. In addition, the number of citizens who voluntarily evacuated to other places while maintaining their residence registry in this area is still unknown, because no accurate records of the population that had moved to different areas are available. There is a great concern that there will be additional health hazards, and we strongly feel the need for administrative support.

Moreover, many medical facilities are unable to reopen because the staff evacuated along with the inhabitants. Patients consequently gather at the few remaining facilities, overburdening the medical staff. An administrative response is also needed for this staffing problem. Based on the experiences from this disaster, medical support teams should attempt to establish a good medical environment not only during the acute period but also based on a long-term perspective.

A number of volunteer doctors came from both Japan and abroad to help and support us. We should assume in advance the type of support required when a disaster happens. If the support stagnates or stops, the on-site staff members will be overworked and possibly suffer from physical and mental diseases themselves, which, in turn, would further disrupt the support system. We sincerely hope our experience will help in the development of medical support systems for future large-scale disasters.

## Conclusion

The coastal area of Fukushima Prefecture was damaged by the Great East Japan Earthquake, tsunami, and accident at the Fukushima Daiichi Nuclear Power Plant, and a large number of people were forced to evacuate. Immediately after the disaster, cases of vertigo and dizziness, MD, and ALHL increased and then slowly decreased over time. There was a simultaneous increase in the number of cases complicated with mental diseases. In treating patients at the time of a disaster, the ENT department needs to understand the patients’ stress and tension over a long period of time and provide psychological care, sometimes working together with psychiatry or psychosomatic medicine departments. The administration has to provide active medical support in response to current disaster situations.

### Ethical approval

This study was approved by the ethics committee of Soma General Hospital, and informed consent was obtained from the subjects.
